# Evaluation of physiological response and synchronisation errors during synchronous and pseudosynchronous stimulation trials

**DOI:** 10.1038/s41598-024-59477-7

**Published:** 2024-04-16

**Authors:** Damian Kania, Patrycja Romaniszyn-Kania, Aleksandra Tuszy, Monika Bugdol, Daniel Ledwoń, Miroslaw Czak, Bruce Turner, Karol Bibrowicz, Tomasz Szurmik, Anita Pollak, Andrzej W. Mitas

**Affiliations:** 1 Institute of Physiotherapy and Health Sciences, The Jerzy Kukuczka Academy of Physical Education in Katowice, Mikołowska 72A, 40-065 Katowice, Poland; 2 Faculty of Biomedical Engineering, Silesian University of Technology, Roosevelta 40, 41-800 Zabrze, Poland; 3 dBs Music, HE Music Faculty, 17 St Thomas St, Redcliffe, Bristol BS1 6JS UK; 4 Science and Research Center of Body Posture, College of Education and Therapy in Poznań, 61-473 Poznań, Poland; 5 Faculty of Arts and Educational Science, University of Silesia, ul. Bielska 62, 43-400 Cieszyn, Poland; 6 Institute of Psychology, University of Silesia, ul. Grazynskiego 53, 40-126 Katowice, Poland

**Keywords:** Health care, Medical research, Engineering

## Abstract

Rhythm perception and synchronisation is musical ability with neural basis defined as the ability to perceive rhythm in music and synchronise body movements with it. The study aimed to check the errors of synchronisation and physiological response as a reaction of the subjects to metrorhythmic stimuli of synchronous and pseudosynchronous stimulation (synchronisation with an externally controlled rhythm, but in reality controlled or produced tone by tapping) Nineteen subjects without diagnosed motor disorders participated in the study. Two tests were performed, where the electromyography signal and reaction time were recorded using the NORAXON system. In addition, physiological signals such as electrodermal activity and blood volume pulse were measured using the Empatica E4. Study 1 consisted of adapting the finger tapping test in pseudosynchrony with a given metrorhythmic stimulus with a selection of preferred, choices of decreasing and increasing tempo. Study 2 consisted of metrorhythmic synchronisation during the heel stomping test. Numerous correlations and statistically significant parameters were found between the response of the subjects with respect to their musical education, musical and sports activities. Most of the differentiating characteristics shown evidence of some group division in the undertaking of musical activities. The use of detailed analyses of synchronisation errors can contribute to the development of methods to improve the rehabilitation process of subjects with motor dysfunction, and this will contribute to the development of an expert system that considers personalised musical preferences.

## Introduction

Sensorimotor synchronisation (SMS) is the coordination of rhythmic movements with an external rhythm, which in its early developments explored broad range of application, from finger tapping to whole musical ensemble’s performance^1^. Tapping fingers in synchronisation with an external rhythm (usually controlled by a computer), often an isochronous metronome, remains a popular paradigm because of its simplicity and long history. The fundamental mechanisms of SMS continue to be investigated through finger-tapping experiments, as the discrete nature of tapping makes the results particularly relevant to measuring reduced variability in auditory-motor performance.

Motor areas of the brain are active when people listen to musical rhythms, even without moving^2^. These facts show that rhythm is an abstract perception that does not depend only on the characteristics of the stimulus or motor responses. Rhythmic structure is a defining characteristic of music^3^. Anticipated beats increase attention to stimuli that generate a certain rhythm, resulting in better discrimination and detection. It can also facilitate movement synchronisation, making it more accurate, less variable and purposeful^4,5^. Beat-based synchronisation can be compared with interval-based synchronisation. This defines the ability to encode and remember the interval between two events, which allows for specific planning of intended actions^6^. In finger tapping studies a series of evenly spaced stimuli are presented, and participant’s task is to simply synchronise with them by tapping their fingers, with a limitation on all other body movement. The literature reveals essential features of human predictive response to a set rhythm which is more accurate with auditory stimuli than for visual stimulation^7^. The synchronisation accuracy is inversely proportional to the time of the applied stimuli. After the stimulus is stopped, a person is able to maintain the correct tempo for many more cycles. Detailed neuroimaging studies have identified a network of auditory and motor structures involved in interval and rhythmic synchronisation^8^. The models developed suggest that motor planning regions play a central role in encoding temporal intervals and providing detailed information to the sensory areas responsible for guiding perception. There are strong relationships between auditory and motor centres, with mechanisms that underlie the synchronisation associated with stimulation from the series of single stimuli.

Auditory-touch stimulation can improve reaction time and stimulus detection—auditory interactions will be particularly evident in the temporal domain, so they play a fundamental role in how an individual interacts with the environment and in music perception^9,10^. The interaction between what one hears and feels is an important aspect of the temporal response to a given auditory stimulus. Research shows that people with musical education (at various levels) show increased cortical signal strength for combined auditory and somatosensory stimuli during stimulation and respond faster to auditory stimuli^11^. There is ample evidence of the functional role of activation of motor areas in the brain in many aspects of auditory perception, not only for movement-related sounds but also during passive listening^12^.

Precise timing is a significant issue in the performance of complex motor sequences stimulated by acoustic stimuli. In these responses, the perception of timing intervals is facilitated by a regular beat in the rhythmic sequence, and individual intervals are encoded relative to this beat or pulse. This phenomenon is termed rhythm-based timing and serves as a time frame for rhythmic entrainment, in which subjects perform specific movements synchronised with the music^13,14^. Rhythmic tapping to an isochronous metronome is the simplest case of entrainment^15^. The ability to perceive the rhythm in music and synchronise body movements with it is defined as beat perception and synchronisation (BPS)^16^.

Asynchrony (synchronisation error) provides fundamental data in any SMS study and is the difference between the time of tapping (contact of the finger with a hard surface) and the time of the corresponding event in an external rhythm^17^. Mean asynchrony is usually negative and is called negative mean asynchrony (NMA)^18^. The rate of external rhythm, usually measured in terms of interonset interval (IOI) duration (or interbeat interval duration, in the case of non-isochronous rhythms or music), is an important independent variable. The capacity for sensorimotor synchronisation takes years to develop. People with motor disorders were found to be impaired in rhythmic tasks, including sensorimotor synchronisation^19,20^. The tapping cycle consists of flexion and extension phases, usually with an immobility phase in between, occurring either at the point of contact (dwell time) or maximum extension (hold time). Pseudosynchronisation, on the other hand, unlike SMS, occurs when participants believe they are synchronising to an externally controlled rhythm but, in fact, control (or produce) the tone with their tapping. In other words, the sound provides auditory feedback on the taps, particularly their tempo and variability^1,21^. The study by Flach et al. noted only a slight and rapid acceleration of tapping immediately after the transition from sensorimotor synchronisation to sensorimotor pseudosynchronisation, regardless of whether participants were informed of the transition^21^. In another study, participants tapped on their own while hearing tap-dependent feedback^22^. It was discovered that participants tapped non-isochronously, partially compensating for the feedback delay, which resulted in more similar (but not perfectly) isochronous feedback tones. These results supported the hypothesis that self-tapping is associated with the timing of integrated sensory (including auditory) consequences of movements. It is also important to verify electromyographic (EMG) patterns during simple movement tasks such as wrist flexion in response to the auditory stimulus^23^.

The potential benefits of using rhythmic auditory stimulation (RAS) in rehabilitating subjects with motor disorders have long been recognised^24^. Hausdorf et al. showed that rhythmic auditory stimulation reduces gait variability in subjects with Parkinson’s disease^25^. Using a metronome also improves the quality of gait when the preset tempo is matched to the preferred one^26^. The use of RAS in the treatment of gait disorders by improving its spatiotemporal parameters in subjects with Parkinson’s Disease has proved useful^27^. The ecological RAS (natural step sounds) and artificial RAS (music-based sounds) contribute to improving biomechanical and clinical gait parameters regardless of the type of stimulation^28^. In a study using auditory stimulation at three set rates, post-stroke subjects showed an increase in step frequency and a reduction in step time of the paralysed side compared to those without auditory stimulation^29^. The study by Ready et al. observed that verifying how gait parameters are influenced by the properties of music (or metronome) and participants’ willingness to engage in musical activities is essential^30^. In the article by Styns et al., participants with varying declared levels of musical training attempted to synchronise their steps to music or a metronome^31^. Participants mostly walked in sync with the beat of the music. Some participants who could not synchronise tended to walk faster when the tempo of the music or metronome was fast. Another study compares the use of music and a simple metronome to synchronise set stimuli with movements during a test of finger tapping, toe tapping and stepping on the spot^32^. Therapeutic interventions using auditory stimulation help improve the motor skills of subjects with gait disorders. It also positively affects their mood, which in the context of neurodegenerative diseases is an extremely important factor^33^. Adaptation, defined as a subject’s ability to observe whole-body movement, can potentially be used for targeted neuromuscular rehabilitation in groups of subjects, depending on the specific treatment goal^34,35^. This is why studying the temporal structure and response to synchronous and pseudosynchronous stimuli is so important as the subject’s response to metrorhythmic stimulation.

## Aim of the study

Based on the literature analysis and the best of the authors’ knowledge, more publications need to compare synchronisation and pseudosynchronisation to inflicted metrorhythmic stimuli to analyse the physiological response of test subjects, considering their background of musical education and sports activity. Therefore, the purpose of the present study was to check the errors of synchronisation and physiological response as a reaction of the subjects to given metrorhythmic stimuli in the context of synchronous and pseudosynchronous stimulation.

The following research hypotheses were proposed:

1. There is a relationship between musical education, musical activity and the values of synchronisation errors.
2. There is a relationship between the values of synchronisation errors and muscles activity parameters, depending on the rate of stimulation.
3. There is a relationship between muscles activity depending on the rate of stimulation.

## Materials and methods

### Participants

Nineteen subjects without diagnosed motor disorders, ranging in age from 18 to 25 years (10 women and 9 men), participated in the study. Measurements were taken in the laboratories of the European HealthTech Innovation Center in Zabrze. Informed consent was obtained from each participant in the study and for publication of identifying information/images in an online open-access publication. Each subject took part voluntarily after being informed of its purpose and procedure. The Bioethics Committee of the J. Kukuczka Academy of Physical Education in Katowice approved the study protocol (No. 1/2023), which the Declaration of Helsinki conducted.

### Research protocol and methods

#### Study 1—pseudosynchronisation during finger tapping test

At the beginning of the experiments, the subjects were asked to fill out a self-administered Music Preference Questionnaire (MPQ) regarding music education, musicianship, involvement in music-related activities and expressed intentions to develop musical skills^36^.

During the relevant part of Study 1, the first stage of the work performed was for the subject to perform a hand lateralisation test. The examinee was asked to spontaneously pick up an object (a pen) in front of them. The hand that performed this was the dominant hand.

**Figure 1:**
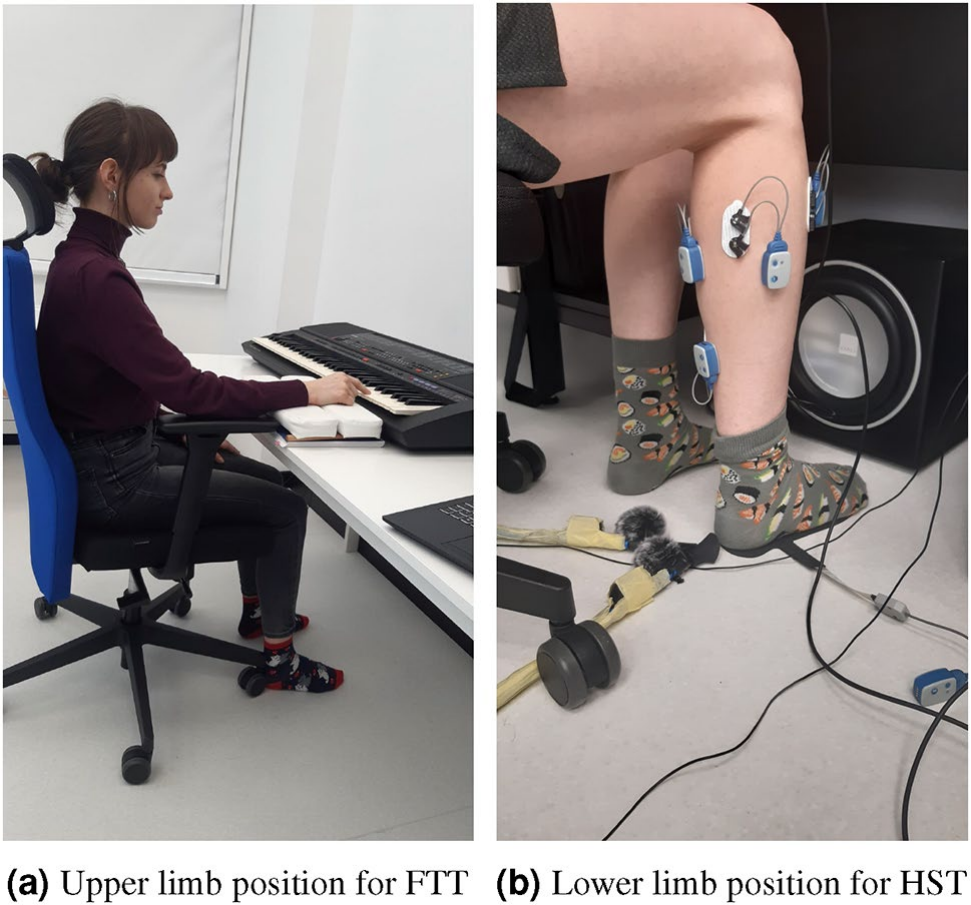
Positions for examination in (**a**) finger tapping test and (**b**) heel stomping test.

Next, the participant was in the correct, well-defined position - sitting on a chair with adjustable seat height and armrests in such a way that the pelvis was supported on the ischial tuberosities, moved to the back edge of the seat, the back straight resting on the back of the chair, and the chest arched forward (Fig. 1a).

The upper limbs had to be positioned with at least a right angle at the elbow joint, the elbows had to be supported on the desk, the arms close to the torso, a gel pad placed in the distal part of the forearm, and the lower limbs supported entirely with the feet on the ground and bent at the knee and hip joints at right angles. After taking the designated place on the dominant hand, an experienced physiotherapist placed four electrodes to measure the electromyographic signal from the appointed muscle parts: extensor digitorum muscle (EDM), palmaris longus (PL), abductor pollicis longus (APL) and flexor digitorum profundus (FDP). These muscles were selected based on the available knowledge of the finger tapping test^37^, which in its original version is the components of the battery of tests for geriatric subject assessment^38^. For this purpose of EMG measurement, a Noraxon Ultium (Noraxon USA Inc., Scottsdale, AZ, United States) system was used, enabling EMG signal recording at 2000 Hz for each measuring electrode independently.

In the next step of the research protocol, the subject performed test taps at the preferred rhythm, on the keyboard pad (Yamaha PSR-500, Yamaha Group, Japan), with the dominant hand’s index finger. At this time, the program determined the frequency, which conditioned the rate of the metrorhythmic beats inflicted in the next step. The concept of preferred rhythm means a comfortable, spontaneous, however constant and repetitive rate of regular beats on the keyboard by the participant.

The following primary step in Study 1 was to perform an adaptation of the finger tapping test (FTT), which involves rhythmically tapping the index finger of the dominant hand on a single, well-defined key on the keyboard, in synchrony with the metrorhythmic stimulus being asked, for a period of one minute. The keyboard was connected via a MIDI-USB adapter to a laptop. The beats reached the subject through professional Beyerdynamic DT 770 PRO headphones (Beyerdynamic GMBH & CO. KG, Germany), and the stimuli were generated using proprietary software that recorded the differences between the generated stimulus and the subject’s response. This test was performed three times, with different modifications of the tempo of the inflicted stimuli. In the first approach, the stimulant was generated according to the rate set during the trial. The second and third approaches successively involved increasing and decreasing the stimulus frequency by 5% relative to the baseline. Changing the stimulation rate by only 5% was decided based on the available literature, which presents the thesis that if the intensity level increases or decreases by a minimum of 10% relative to that chosen by the exerciser, it reduces the sensation of pleasure^39^.

#### Study 2—synchronisation during heel stomping test

The next elements of the research protocol, the exercise included in Study 2, looked similar to the previous one. First, the lower limb lateralisation test was performed. The physiotherapist conducting the test, while protecting the test subject (holding him from behind by the shoulder), gently pushed him. The first leg that the test person took a step forward was the dominant leg. The subject then took a position on a chair similar to that of Study 1 (Fig. 1b).

The physiotherapist, in the next step of the research protocol, placed EMG measuring electrodes, on the dominant lower limb, on strictly defined parts of the muscles: gastrocnemius lateralis (GL), gastrocnemius medialis (GM), soleus (SL) and tibialis anterior (TA). The GL, GM, SL, and TA muscles are important in providing proprioceptive information and muscle agonist function necessary to maintain a person’s upright vertical posture and stability^40^. They play primary roles during active plantar flexion and dorsiflexion of the foot. The main plantar flexors of the foot are the GL, GM and SL muscles, with the SL muscle playing a more important role during plantar flexion of the foot when the knee is bent at 90 degrees. In the standing position, the TA muscle seems to be a better source of proprioceptive information compared to the agonist muscles GL, GM and SL. An appropriately sized Noraxon Ultium insole was then placed under the foot to record the ground reaction forces at 2000 Hz (the moments the heel taps the ground and the force of the tapping). At the back, behind the heel, were two sensitive SYNCO LAV-S6 R microphones, which recorded the sound generated when the foot tapped. The proband’s ears were fitted with Beyerdynamic DT770 Pro 80 Ohm headphones connected to a smartphone running a metronome app to audition sonic beats. In addition, a second pair of headphones was hooked up to a smartphone connected to an all-sound recording device, the ZOOM H4n Pro, which allowed 4-channel recording (two for microphones at the foot, two for metronome sounds). The subject was then asked to freely tap his/her heel on the ground at his/her preferred pace for about 30 s.

In the next step of the research protocol, the subject’s task, similar to that of Study 1, was to try to synchronise heel taps against the ground with the rhythm of the inflicted metrorhythmic stimulus at a predefined and personalised rate, i.e. to perform the so-called heel stomping test (HST). This test was also performed three times at different rates of metrorhythmic stimulus generation: (1) basal, (2) accelerated by 5%, and (3) delayed by 5% relative to the initial one. Each time, the EMG signals, the metronome and the recorded sound were independently synchronised.

The figure below shows the positions of the subjects during each test (Fig. 1).

### Data preprocessing

#### Finger tapping test

**Figure 2:**
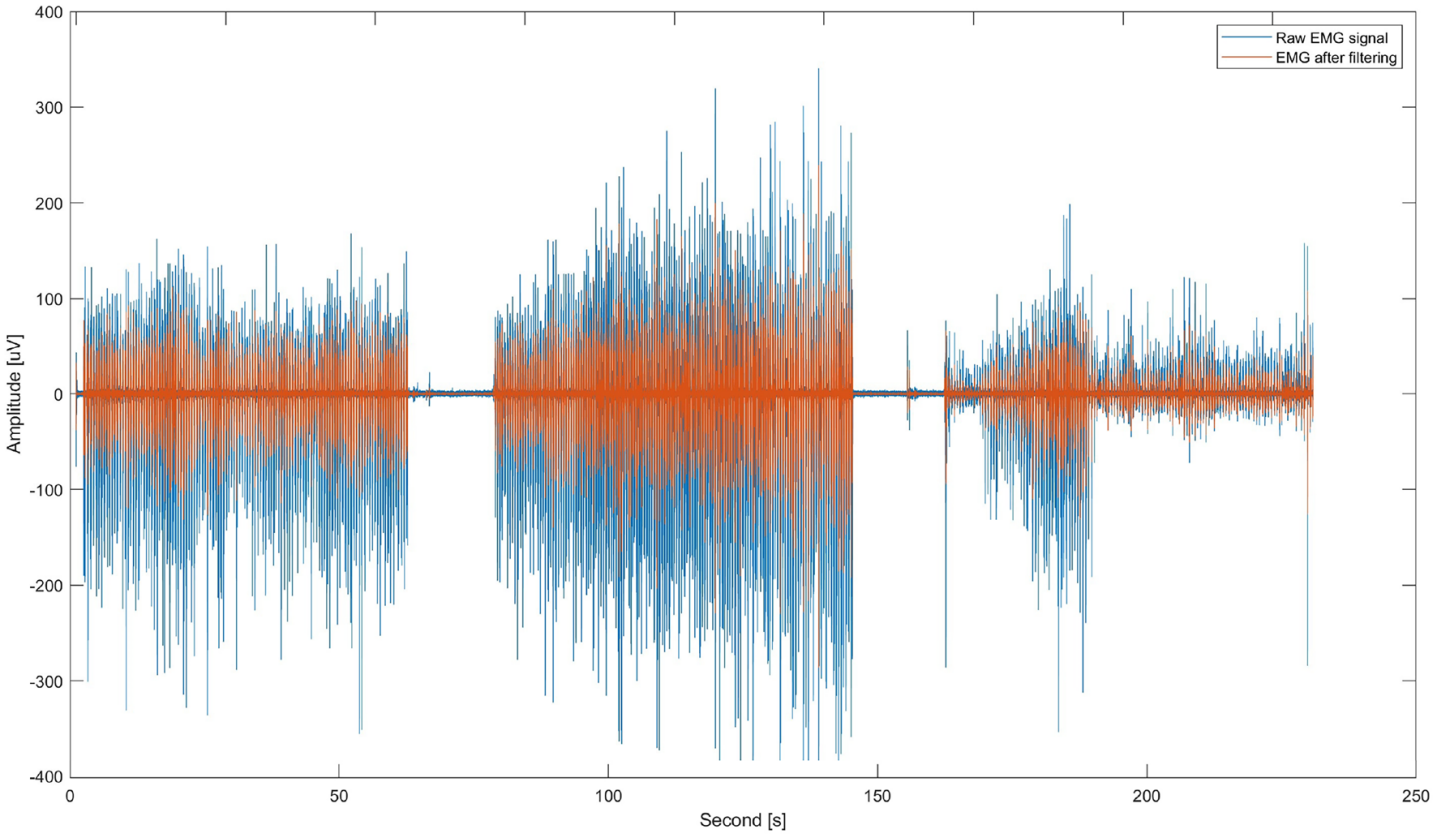
Raw (blue) and filtered (orange) EMG signal recorded during study 1 from palmaris longus muscle.

In the first step, EMG preprocessing was performed, for each muscle in the same way, based on the marked time markers of the beginning and end of each test step (Fig. 2). For this purpose MATLAB R2023b was used.

**Figure 3:**
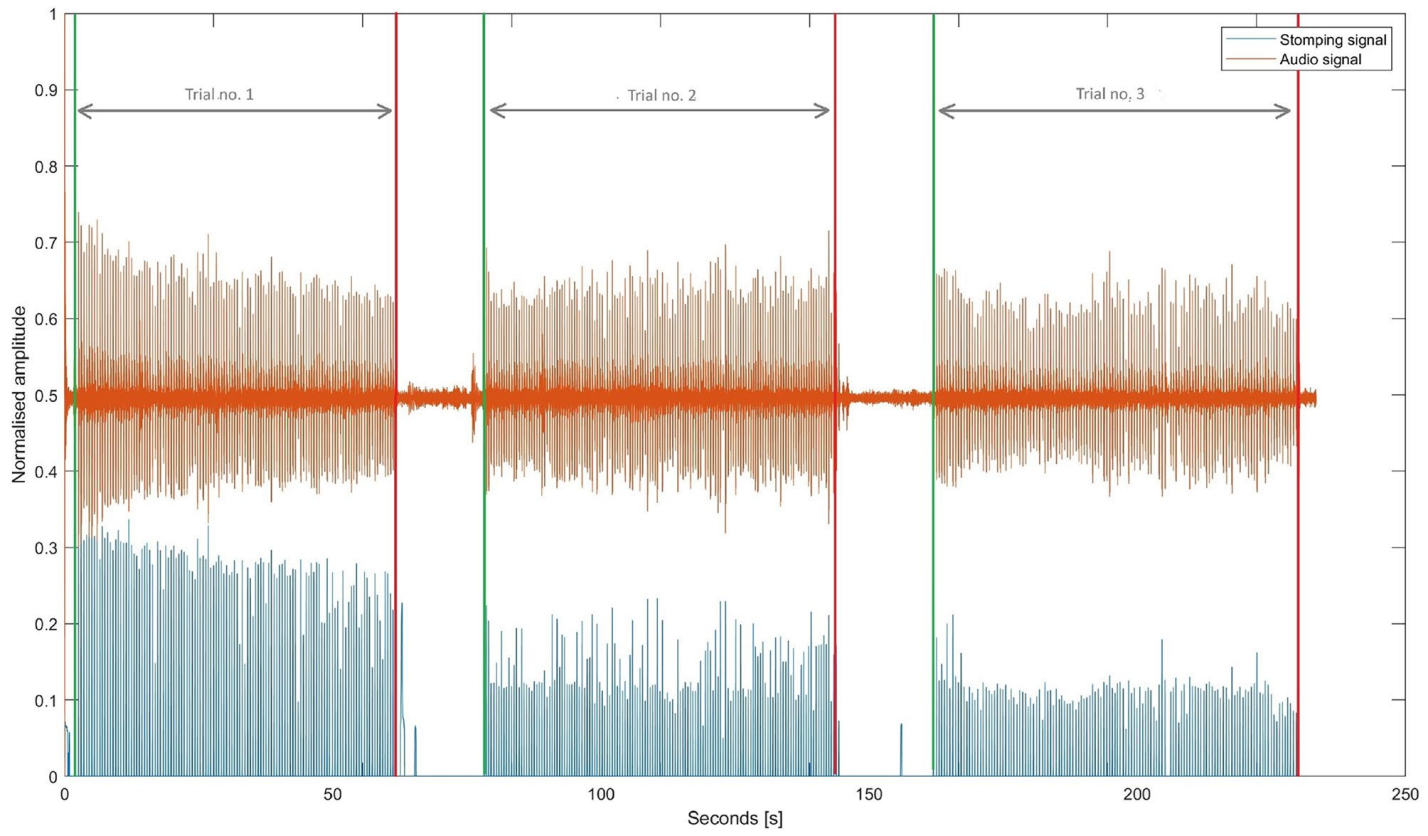
Metoronome’s audio signal (orange) and tapping signal (blue) divided into trials.

The EMG signal was brought to the baseline and filtered with a 50 Hz high-pass filter and a 60 Hz low-pass filter according to the available literature^41^. In the next step, the envelope of the EMG signal was determined, and based on the given rate of excitation and the hypothetical minimum distance between successive movements, the stages of contraction and diastole of the muscle were calculated. This allowed to calculate the average values of signal characteristics in a given time frame, such as the maximum value, the time after which maximum contraction force was reached, the mean value of contraction, variance, skewness, kurtosis, Mean Absolute Deviation (MAD), Waveform Length (WL), Wilson Amplitude (WA), Coefficient of variation (CoV), average signal frequency and frequency response, and power density ratio (PDR)^42^. Linear regression coefficients were also determined for the following characteristics in successive contractions of individual muscles: maximum tension, mean value and time to maximum. Next, the latency or acceleration times were calculated, i.e., the synchronisation errors between the subject’s successive responses to a given metrorhythmic stimulus (key press) and the moments of stimulus onset (Fig. 3).

From the results obtained, timeout occurrences were removed and features such as mean reaction time (synchronisation error), median, standard deviation, variance, skewness, kurtosis, minimum and maximum value were determined. In addition, the differences between successive synchronisation errors, their mean, minimum, maximum, median and standard deviation were calculated.

#### Heel stomping test

Multimodal data were recorded during the second part of the research protocol. First, the audio, EMG and force signals were synchronised by the first more robust heel-to-floor tap, which produced a distinct change in amplitude value in each recorded signal. This also made it possible to isolate the different stages of the test related to the change in the rate of the stimuli, which were processed analogously. After the signals were trimmed appropriately, the audio signal was downsampled, from 44100 Hz to the EMG signal/pressure force sampling frequency 2000 Hz, without losing any relevant information. Then, based on the audio signal of the metronome and the force signal, or the moments of heel-to-toe contact from the insole, the lag/acceleration time between successive heel taps and the beat sound was calculated as the difference in the number of samples between the closest local maxima, or synchronisation error. Preprocessing of the EMG signal recorded during this step (Fig. 3) was conducted in the same way as in Study 1. The analysis of ground reaction forces (recorded from an insole placed under the foot of the dominant limb) was excluded as it was not essential to the presented work.

The conducted process of analysing questionnaire methods, results were obtained from a music preference survey, which was analysed without an imposed key. Each question represented independent information about the individual regarding social and economic elements. Based on the survey responses, it was decided to distinguish two groups of respondents in terms of (1) having any experience of music education and having no experience of music education at all, (2) taking up or not taking up music activities, and (3) taking up or not taking up sports activities. Interrupted music schooling was included in the division because the respondents declared that they were undertaking additional activities, such as systematically taking instrument lessons or participating in extracurricular activities related to music.

### Statistical analysis

Comparisons of two independent samples were performed with the Student’s t-test for unpaired samples or the U Mann-Whitney test, depending on the fulfilment of the t-test’s assumptions (normality-Shapiro-Wilk test, variance homogeneity-F test). A statistically significant effect was assessed in terms of its size employing Cohen’s d or Glass rank biserial correlation coefficient, respectively. Comparisons of three paired samples were performed using ANOVA for paired samples or the Friedman test when the assumptions of normality or sphericity (Mauchly test) were not met. The effect size was estimated employing eta-squared or Kendall’s W, respectively. Correlations were estimated with the Pearson correlation coefficient. Small effects are not reported in this study, since they have no clinical impact. The significance level was set to alpha=0.05. The analysis used R 4.3.1 and RStudio 2023.06.1+254 with packages tidyverse, car, rstatix, and rcompanion.

## Results

### Finger tapping test

**Figure 4:**
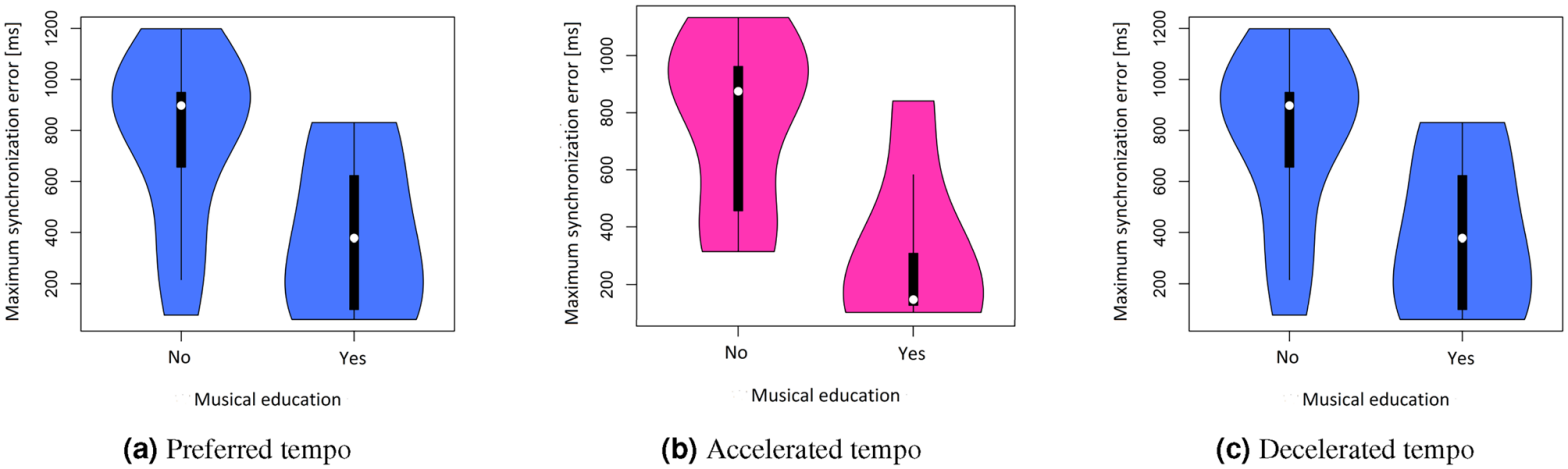
Statistically significant parameters of synchronisation error differences.

No statistically significant differences were found for any of the three grouping variables—general synchronisation error depending on music education, music activities and sports activities.. Statistically significant differences were obtained between subjects with and without musical education for the maximum values of time differences in all three trials (Fig. 4). Musical education was significant for the differences in the subsequent synchronisation errors of the group of subjects in the first trial ($p=0.0257$), second trial ($p=0.0039$) and third trial ($p=0.0184$) relative to the maximum delay differences.

Dividing the subjects by musical education, the linear regression coefficient of the time to maximum single contraction during a finger tap on a keyboard key, on the first trial, was significantly different for the abductor pollicis longus muscles ($p=0.043$). Respondents with musical education took longer to reach maximum contraction, while those without had less time.

Statistically significant differences were found for the mean maximal contraction value of the palmaris longus muscle for those declaring and undeclaring musical activity ($p=0.0025$). Using a preferred tempo of metrorhythmic stimulation, those reporting musical activity achieved a significantly higher mean value than the inactive group. Similar results were conducted for this muscle in the accelerated pace trial ($p=0.022$) and in the slower rate trial ($p=0.0187$). The skewness of the EMG signal for different rates of stimulation for the palmaris longus muscle also differentiated the group of subjects in terms of undertaking musical activities (trial 1 $p=0.0435$, trial 2 $p=0.0431$, trial 3 $p=0.0279$). For the same muscle, for musically active subjects, the EMG signal showed significantly lower within-group variation based on CoV values, compared to inactive subjects, for each successive trial (trial 1 $p=0.0172$, trial 2 $p=0.0133$, trial 3 $p=0.035$). There was also significantly higher variability between EMG signal amplitudes (Willison amplitude) during successive finger taps on a second trial ($p=0.0485$) for the same muscle in the musically active group. A measure of variability in EMG values in the form of the MAD parameter was significantly higher in the musically active group ($p=0.0292$), in the third trial, with a delayed tempo. In the case of dividing the group of subjects in terms of sports activities performed or not, a significantly higher value of the average fundamental frequency of the EMG signal recorded from the palmaris longus muscle during FTT was obtained in the sports-active group in the first ($p=0.0062$), second ($p=0.0133$) and third trial ($p=0.0101$). Detailed results (mean value ± standard deviation) are shown in Table 1.

**Table 1:** Mean values of individual significant EMG signal parameters in FTT with statistically significant values marked (* p<0.05, ** p<0.01, *** p<0.001).

			Trial 1	Trial 2	Trial 3
Music education	APL_regression_timeMax	Yes	0.000349 (0.000756)*	−0.00031 (0.000504)	0.00021 (0.000488)
		No	−0.00024 (0.000439)*	−0.00035 (0.000673)	−0.00071 (0.002)
Music activity	PL_max [uV]	Yes (n=10)	253.29 (165.19)**	214.13 (93.83) *	253.43 (156.80)*
		No (n=9)	96.58 (52.8)**	119.65 (68.85)*	98.86 (46.68)*
	PL_skew	Yes	2.13 (0.58)*	2.12 (0.52)*	2.16 (0.56)*
		No	1.72 (0.31)*	1.71 (0.27) *	1.75 (0.39)*
	PL_CoV	Yes	1.24 (0.35)*	1.14 (0.18)*	1.20 (0.23)*
		No	0.97 (0.09)*	0.98 (0.08)*	1.02 (0.17)*
	PL_WA	Yes	1017.66 (406.50)	975.11 (276.60)*	921.95 (242.52)
		No	950.27 (186.73)	970.77 (172.45)*	899.09 (142.02)
	PL_MAD	Yes	10.87 (6.64)	8.45 (5.88)	11.65 (6.23)*
		No	5.98 (3.49)	8.81 (5.29)	6.18 (3.58)*
Sport activity	PL_F0	Yes	39.92 (1.72)**	39.77 (1.84)*	40.00 (1.82)*
		No	37.73 (1.28)**	37.76 (1.12)*	38.02 (1.22)*

*APL* abductor pollicis longus, *PL* palmaris longus, *CoV* coefficient of variation, *WA* Wilson amplitude, *MAD* mean absolute deviation, *F0* fundamental frequency.

Statistically significant differences were obtained between successive trials for the WA parameter for the palmaris longus muscle ($p=0.0028$) and for the abductor pollicis longus muscle ($p=0.0006$), as well as for the PDR parameter of the abductor pollicis longus muscle ($p=0.0083$).

**Figure 5:**
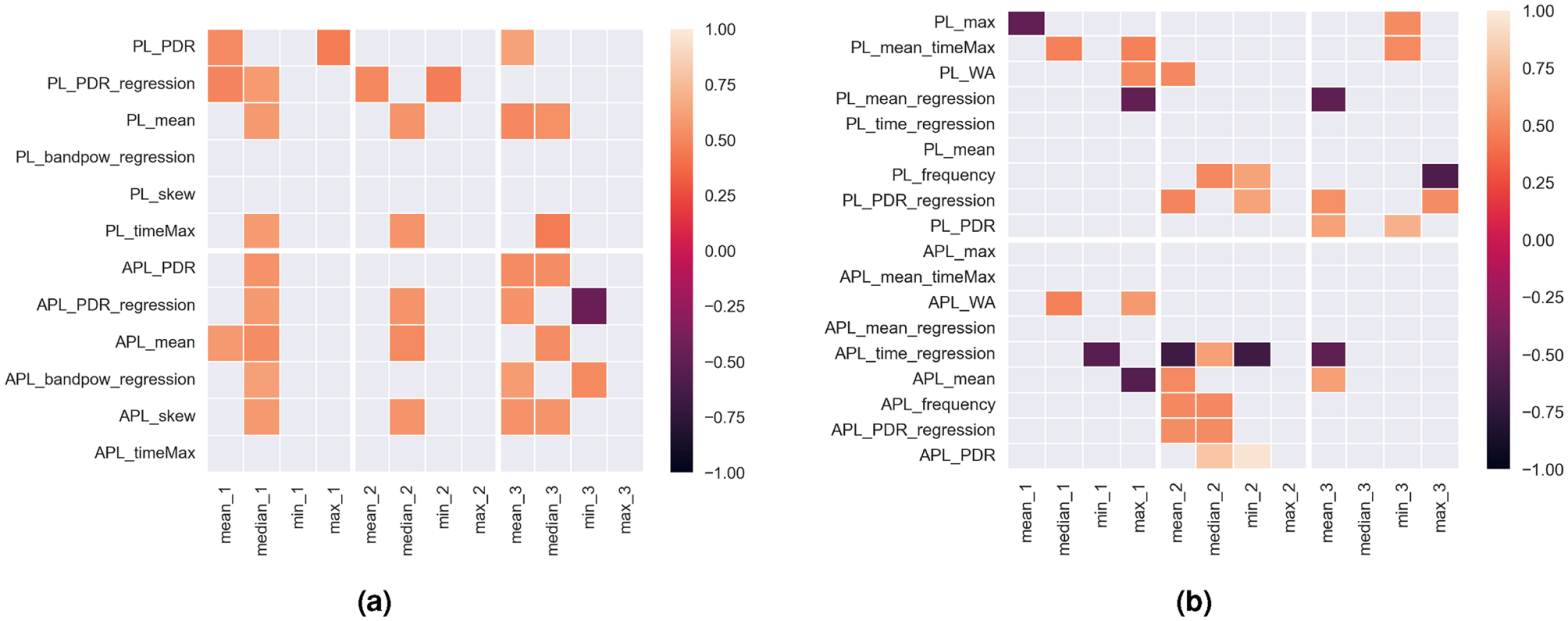
Statistically significant correlation coefficients of EMG parameters with (**a**) reaction time and (**b**) parameters of differences between successive synchronisation errors during FTT.

Statistically significant correlation coefficients *r* ($p<0.05$) between reaction times and EMG signal features in succesive trials are shown in Fig. 5a. The significant correlation coefficients between the differences determined for successive synchronisation errors and EMG signal parameters for individual muscles in subsequent trials are shown in the following figure (Fig. 5b).

**Figure 6:**
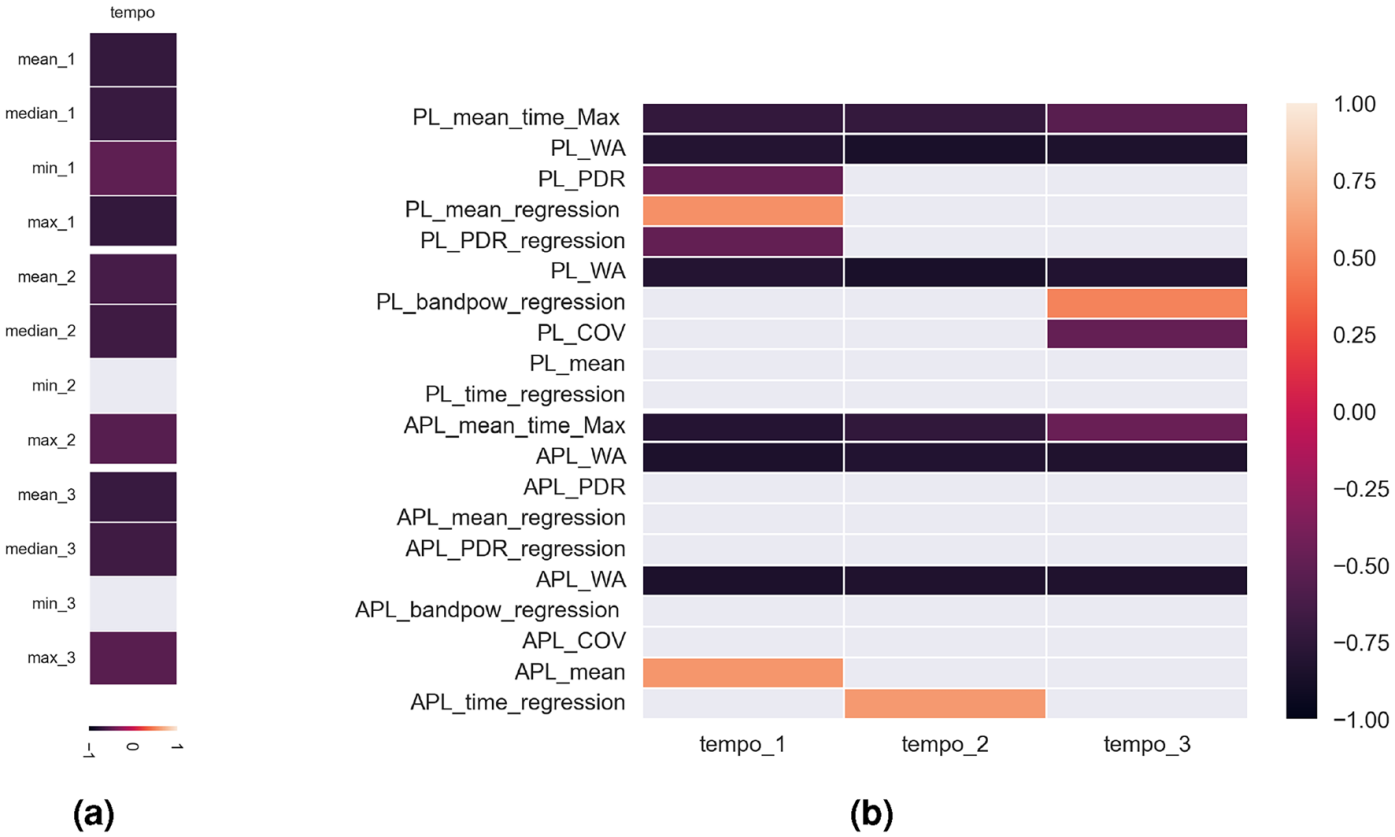
Statistically significant correlation coefficients of pseudocynchronous stimulation rate with (**a**) EMG parameters and (**b**) time differences between successive sychronisation errors during FTT.

Significant correlations were obtained between the tempo of the stimulus in trial one and the minimum value of the difference in time between synchronisation errors ($r=-0.51$), the maximum value ($r=-0.73$), the mean ($r=-0.72$), and the median ($r=-0.7$). For the second trial, there were significant correlations between rate and maximum value ($r=-0.55$), mean ($r=-0.63$), and median ($r=-0.67$). For trial three, significant correlations were determined between pace and maximum time difference ($r=-0.54$), mean ($r=-0.7$), and median ($r=-0.67$) (Fig. 6a). In the last step of the performed analysis for Study 1, it was also checked whether, and if so, with which coefficients of the EMG signal for individual muscles of the upper limb correlate the value of the set rate during subsequent FTT trials. The significant correlation coefficient values between EMG coefficients for individual muscles of the upper limb and the tempo during subsequent trials are shown in Fig. 6b.

### Heel stomping test

**Figure 7:**
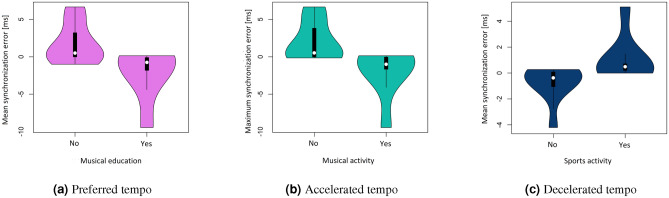
Statistically significant mean values of synchronisation errors with respect to individual differential variables during HST.

The mean reaction time significantly differentiated the test group in tests with different tempo of stimulus (Fig. 7). Musical education and musical activity significantly determined the reaction time of the group of subjects in the first trial ($p=0.0075$ and $p=0.0079$ respectively). At slowed rates, significant differences in synchronisation errors were obtained relative to the sports activity ($p=0.0115$).

The difference in times between successive synchronisation errors significantly differentiate the group of subjects regarding musical education, musical activity and sports activity. In the case of musical activity, the minimum values of the difference in the times of successive synchronisation errors in the first trial ($p=0.0247$) and in the second trial ($p=0.0022$) were significantly different. The maximum value in the first trial of differences in times of consecutive synchronisation errors ($p=0.0115$), mean ($p=0.0164$), and median ($p=0.0418$) significantly differentiated the group in terms of declared sports activity.

Subjects declaring musical education showed a significantly different time to reach the maximum value of a single muscle contraction during successive repetitions of the HST, on a trial-by-trial basis, compared to subjects without musical education. The music group generated a decreasing maximum contraction of the Gastrocnemius medialis muscle in trial 3 ($p=0.0223$) and an increasing maximum contraction of the soleus muscle in trial 2 ($p=0.0075$). In successive repetitions the musically educated decreased the mean muscle tension in the Gastrocnemius medialis muscle in trial 3 ($p=0.011$) and significantly increased it in trial 2 in the soleus muscle ($p=0.042$). Musically active respondents showed significantly higher mean maximal muscle tension values in trial 2 ($p=0.0347$) in the Gastrocnemius lateralis muscle. The change in these values in successive taps during a single trial was also significantly different in the comparison group of subjects. Musically inactive subjects showed an upward trend in the maximum value in trial 3 for the Gastrocnemius lateralis muscle ($p=0.0052$) and a downward trend in trial 2 for the soleus muscle ($p=0.0202$). The trend of the mean value of muscle tension in subsequent trials was also significantly different. For the Gastrocnemius lateralis muscle, musically active subjects showed a decrease in the mean value in trial 3 ($p=0.0311$) and an increase in trial 2 for the soleus muscle ($p=0.0229$). The PDR parameter values were significantly higher for the musically inactive group in sample 2 ($p=0.0418$) and sample 3 ($p=0.0418$) for the Gastrocnemius lateralis muscle and the Gastrocnemius medialis muscle in sample 3 ($p=0.0021$). In the case of those declaring sports activity, a higher mean value of muscle tension in trial one was obtained for the Gastrocnemius lateralis muscle ($p=0.0418$). There was also a significantly higher value of the WA parameter between EMG signal amplitudes during successive foot taps, during trial three for the Gastrocnemius lateralis muscle ($p=0.0311$) and trials two ($p=0.0418$) and three ($p=0.0311$) for the Gastrocnemius medialis muscle, in the group of sports-active individuals. The skewness of the EMG signal for different rates of stimulation for the Gastrocnemius medialis muscle also differentiated the group of subjects in terms of undertaking sports activities in trial one ($p=0.0442$), as well as trial two for the soleus muscle ($p=0.0148$). There was a right-sided asymmetry in the distribution each time, indicating that most of the recorded EMG signal values were below the mean. Athletically active subjects achieved a significantly shorter time in a single foot tap to generate the maximum muscle tension value in the Gastrocnemius medialis muscle in trial 3 ($p=0.0418$) and trial 2 for the soleus muscle ($p=0.0418$) (Table 2).

**Table 2:** Mean values of individual significant EMG signal parameters in HST with statistically significant values marked (* p<0.05, ** p<0.01, ** p<0.001). .

			Trial 1	Trial 2	Trial 3
Music education	GM_regMax	Yes	−0.05 (0.66)*	−0.28 (0.67)	−0.56 (0.7) *
		No	0.01 (0.46) *	0.04 (0.41)	0.19 (0.47) *
	GM_reg_mean	Yes	−0.01 (0.06) *	−0.05 (0.1) *	−0.13 (0.15)*
		No	−0.01 (0.06) *	0 (0.08) *	0.03 (0.08)*
	SL_time_regression	Yes	0.09 (0.19) *	26.95 (65.55)*	−0.3 (0.31) *
		No	0.13 (0.67)*	−7.1 (22.26)*	−0.16 (0.3) *
	SL_mean_regression	Yes	0.01 (0.04) *	3.16 (7.74) *	−0.03 (0.04) *
		No	0.01 (0.13) *	−0.09 (0.24) *	−0.03 (0.06) *
Music activity	GL_max	Yes	448.89 (441.06)	550.84 (340.4)*	379.82 (331.4)
		No	270.03 (226.62)	250.28 (175.75) *	246.58 (192.16)
	GL_reg_max	Yes	0.41 (0.73)	0.08 (0.29)	−0.4 (0.56)**
		No	0.17 (0.48)	0.23 (0.41)	0.56 (1.03)**
	SL_reg_max	Yes	0.09 (0.17) *	23.11 (60.7)*	−0.24 (0.32) *
		No	0.14 (0.71) *	−7.89 (23.46)*	−0.19 (0.3)*
	GL_mean_regression	Yes	0.07 (0.14)	0.01 (0.05)	−0.07 (0.09)*
		No	0.02 (0.06)	0.05 (0.09)	0.09 (0.18) *
	SL_mean_regression	Yes	0.01 (0.04)	2.71 (7.17) *	−0.03 (0.04) *
		No	0.02 (0.14)	−0.1 (0.26) *	−0.03 (0.06) *
	GL_PDR	Yes	6.53e8 (1.44e9)	2.69e8 (5.22e8) *	9.36e8 (4.34e7) *
		No	9.16e8(1.43e9)	2.42e9 (6.16e9) *	4.34e8 (5.94e8)*
	GM_PDR	Yes	6.56e8 (1.16e9)	8.66e8 (6.33e8 )	4.26e8 (3.82e8 )**
		No	1.24e9 (2.05e9)	3.18e8 (3.97e8 )	1.69e8 (1.51e8 )**
Sport activity	GL_mean	Yes	0.03 (0.33)*	0.01 (0.07)	0.06 (0.11)
		No	0.05 (0.08) *	−0.01 (0.27)	−0.03 (0.2)
	GL_WA	Yes	523.07 (141.62)	593.26 (202.98)	657.55 (244.59)*
		No	454.99 (145.42)	519.81 (455.85)	433.61 (123.82)*
	GM_WA	Yes	632.35 (374.54)	552.77 (325.31) *	618.13 (241.91) *
		No	363.25 (184.87)	335.78 (107.84)*	370.35 (192.57) *
	GM_skew	Yes	0.03 (0.07) *	0.01 (0.09)	0.03 (0.06)
		No	−0.08 (0.11)*	−0.07 (0.13)	−0.04 (0.08)
	SL_skew	Yes	1.63 (0.32)	1.69 (0.28)*	0.04 (0.12)
		No	1.54 (0.41)	1.37 (0.76) *	0 (0.04)
	GM_timeToMax	Yes	0.14 (0.09)	0.12 (0.09)	0.13 (0.07) *
		No	0.08 (0.03)	0.08 (0.02)	0.08 (0.04) *
	SL_time_regression	Yes	0 (0)	0 (0)*	0 (0)
		No	0 (0.01)	−0.27 (0.81) *	0 (0)

*GM* gastrocnemius medialis, *SL* soleus, *GL* gastrocnemius lateralis, *PDR* power density ratio, *WA* Wilson amplitude.

It was shown that a statistically significant difference exists only for the frequency parameter determined from the EMG signal of the Gastrocnemius lateralis muscle ($p = 0.008$) and for the CoV parameter of the soleus muscle ($p=0.0072$).

**Figure 8:**
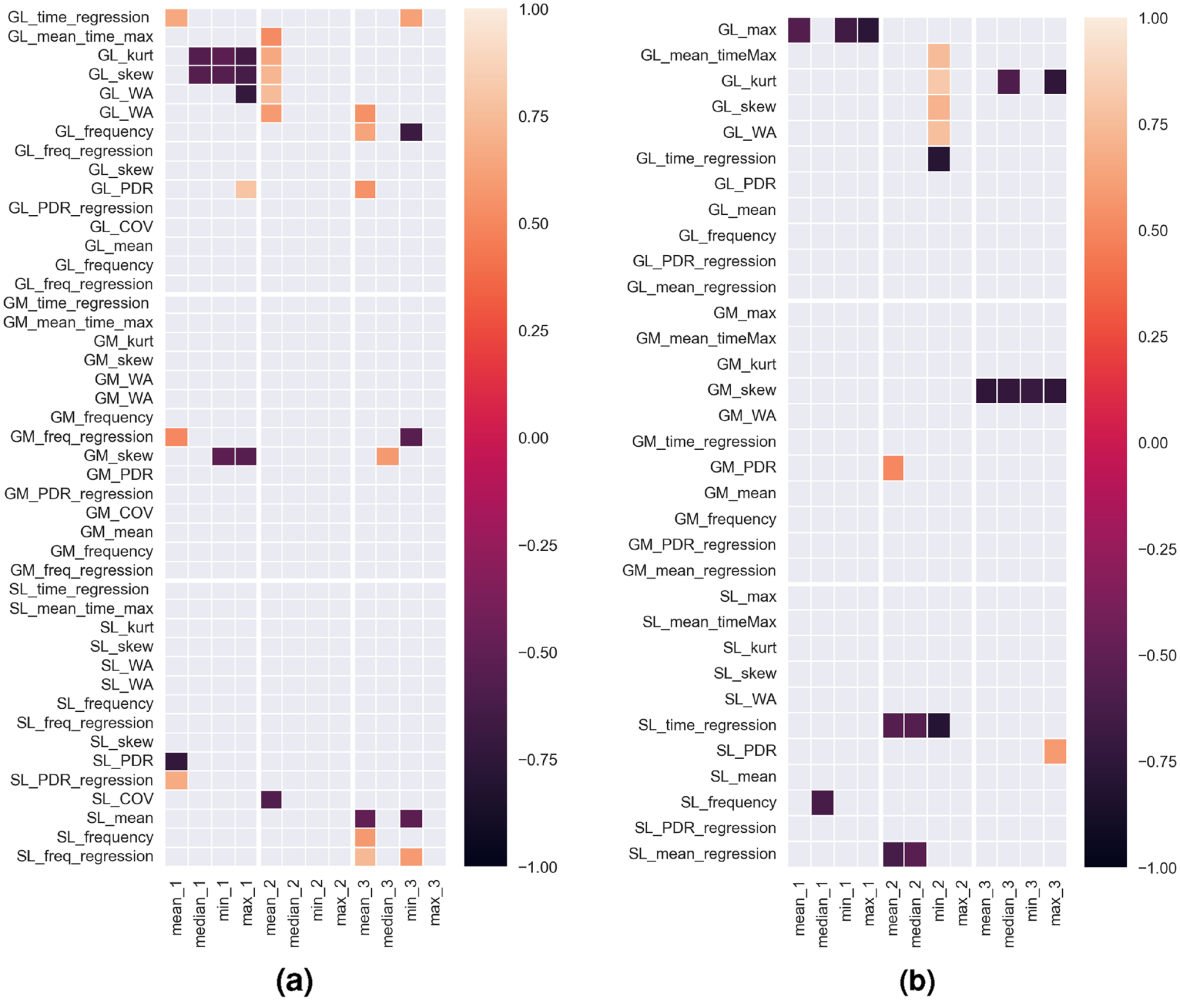
Statistically significant correlation coefficients of EMG parameters with (**a**) reaction time and (**b**) parameters of differences between successive synchronisation errors during HST.

Statistically significant correlation coefficients between synchronisation errors and EMG features are shown in Fig. 8a. It was verified and proved that there are also statistically significant correlations between the parameters of time differences in synchronisation errors and the parameters of the EMG signal from individual muscles in successive trials (Fig. 8b). The following results present all the variables that were included in the analyses conducted, allowing other researchers to make comparisons with our results (reproducibility).

There were no statistically significant correlations between the parameters of reaction time and the tempo of synchronous metrorhythmic stimuli in successive trials and between the parameters of time differences of consecutive synchronisation errors and the given tempo.

**Figure 9:**
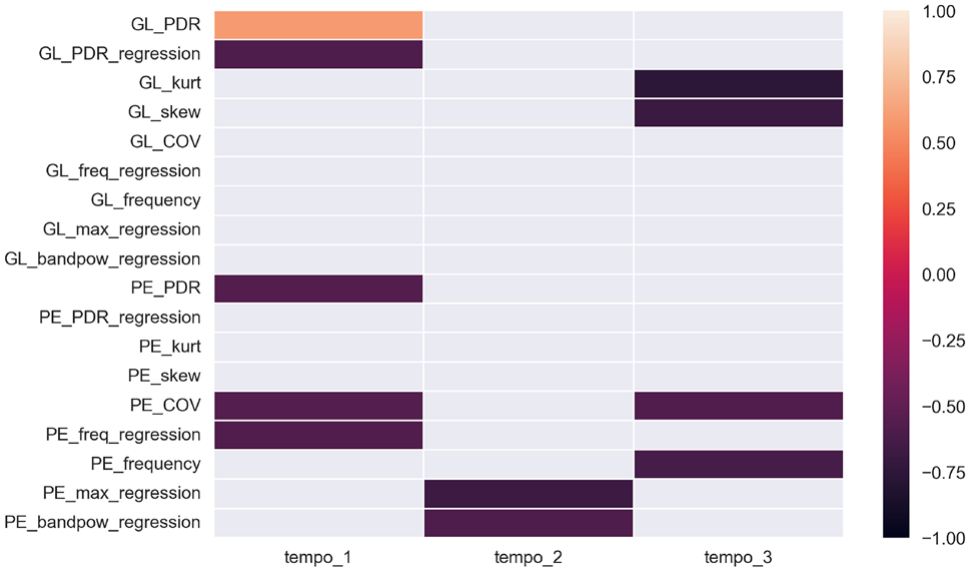
Statistically significant correlation coefficients of the rate of stimulation given metrorhythmic stimuli with EMG signal parameters for all muscles, in consecutive trials.

The final stage of the analysis involved checking the correlation between the rate of stimulation of the metrorhythm and the EMG parameters in a given trial. Statistically significant correlation coefficients are shown in the following figure (Fig. 9)

## Discussion

Many scientific reports have investigated the relationship between music and extra-musical abilities^43^. Sound is a common phenomenon perceived by humans continuously, which in an orderly form, induces changes at the physiological and behavioural levels^44^. It stimulates the limbic system in the brain, including the amygdala, which is responsible for producing physiological responses to external stimuli^45^. Rhythmic coordination of movements with the inflicted external rhythm in the form of sensorimotor synchronisation or pseudosynchronisation are musical abilities that have a neural basis, and the so-called asynchrony, or errors of synchronisation, allow an analysis of the body’s response to applied stimuli^46^. Particular attention is paid to synchronisation errors in auditory stimuli to improve subjects with body motor disorders. Therefore, in the presented article, it was decided to analyse synchronisation errors and physiological response as a reaction to inflicted metrorhythmic stimuli, in the context of synchronous and pseudosynchronous stimulation, depending on external factors.

As part of the ongoing work, subjects performed a finger tapping test during a pseudosynchronous stimulation trial. There were no statistically significant differences in synchronisation errors for any of the three grouping variables (due to musical education, musical and sport activity).

An analogous analysis was performed for synchronous stimulation using the heel stomping test. In this approach, the mean values of synchronisation errors differentiated the group by musical education (trial one), musical activity (trial one) and sports activity (trial three). Those declaring themselves musical performed a heel tap before the onset of the stimulus in the first trial, while in the third trial, where the stimulus infliction rate was 105% of the baseline rate, the sport-active subjects accelerated relative to the onset of the sound. The results illustrate that the occurring mean values of synchronisation errors did not reach high values, which may indicate the subjects’ high concentration during the HST performance. Confirmation of this phenomenon is not found in the literature. According to a study by Repp et al., subjects described as “nonmusicians” performed taps preceding the corresponding tone during the isochronous tone sequence tap test^47^. However, the differences may be because the group of surveyed ’musicians’ in the above mentioned article were long-term, experienced professional musicians and not, as in our article, only with musical training and were not currently practising. Also, in the case of time differences occurring between successive synchronisation errors, musical activity (trials 1 and 2) and sports activity (trial 1) significantly differentiated the group of subjects. While these differences were insignificant in the FTT, subjects not declaring the activity in question scored several times higher during the HST. This test for the subjects was probably related to the more natural movement they perform more often than toe tapping, if only when listening to music in line with their preferences or performing a particular sports activity. Achieving smaller values of minimum, average and maximum differences between successive synchronisation errors may indicate better perception of sounds and more developed hearing in the case of active people. The process of musical training and subsequent experience in this field determines the ability and sensitivity to auditory stimuli, so it is different in people without musical training^48^. Developed hearing skills, including analytical hearing, is another characteristic that, according to literature reports, can distinguish the musically educated from the musically uneducated, which is directly reflected in the results obtained in our study^49^. A study by Landry et al. shows that musically educated individuals have significantly shorter reaction times to both auditory stimulus and tactile and auditory-tactile stimulation^50^. This is also supported by a study by Brochard et al. in which reaction time to an emerging visual stimulus was significantly shorter in a group of musicians^43^. A study by Scheurich et al. showed that a group of musicians synchronised more flexibly across tempos than non-musicians, as indicated by higher synchronisation accuracy. In addition, musicians showed greater involvement in error correction mechanisms than non-musicians^51^. The differences in synchronisation errors obtained in our study support this conclusion. Repp’s study also indicated, on average, lower asynchrony among musicians, lower tapping variability and greater perceptual sensitivity than non-musicians^52^. Musical education significantly differentiated the group of subjects regarding the time regression to reach the maximum contraction of the pollicis longus muscle. Subjects declaring musical education needed more and more time to reach this state, which may indicate less muscle fatigue, endurance and probably a peculiar adaptation to the movement performed within the FTT due to the analogy with playing instruments. Based on a variable also related to the subjects’ musicality—musical activity—significant features were extracted in each successive FTT trial. Musically active subjects generated higher maximum tension values during the palmaris longus muscle contraction. The skewness of the EMG signal for the same muscle each time showed a significant rightward asymmetry of the distribution, indicating that most of the recorded EMG signal values in the active group are below the mean value. Significantly lower intragroup variation was also obtained for the active, indicating the results’ reproducibility and the similarity in the physiological response of musically active subjects. Considering the sports activity of the group, the variation was only for the fundamental frequency of the EMG signal also for the palmaris longus muscle. Based on the presented results, it can be assumed that the performance of the FTT test made it possible to isolate groups of significant EMG signal parameters with different information-carrying capacities, differentiating hypothetical subjects depending on the adopted differentiating variable, and indicates the significance of only one of the four analysed muscles of the upper limb—the palmaris longus muscle. Musical training is a feature of analysing changes in the EMG signal over time. Musical activity can be described by analysing typically statistical features, while for sports activity, the frequency features of the signal may be crucial. In the case of the HST test, those with musical training generated a decreasing maximum value for the Gastrocnemius medialis muscle in trial 3 and an increasing value for the soleus muscle in trial 2. The average value of muscle tension also decreased. The difference between the consecutive values for the Gastrocnemius medialis muscle in trial 3, and the indicated parameters for the soleus muscle in trial 2 increased significantly. This is directly reflected in the rate of inflicted excitation—the average and maximum muscle tension value depends on the interval between successive contractions. Increasing this interval causes a decrease in the strength of contraction and its shortening—an increase in the strength of contraction^53^. Similar results were obtained for the division of the study group concerning undertaking musical activity. This confirms the fact that the response of the subjects in contact with music is the literature pattern of the physiological response of the body to the inflicted stimulus, and the essential parameters distinguishing the musical and non-musical groups are the basic statistical coefficients of the EMG signal, which directly reflect the instantaneous state and change in muscle tension. It has also been proven that only two (GL, PE) of the four analysed lower limb muscles have the highest informative value and reflect the physiological state of the subjects tested for musicality. . They play primary roles during active plantar flexion and dorsiflexion of the foot. The main plantar flexors of the foot are the GL, GM and SL muscles, with the SL muscle playing a more important role during plantar flexion of the foot when the knee is bent at 90 degrees. In the standing position, the TA muscle seems to be a better source of proprioceptive information compared to the agonist muscles GL, GM and SL. According to literature reports, groups of musicians and non-musicians differ in many aspects - both neurobiological and electrophysiological^54^. Reaction time (synchronisation error values) during the FTT test was significantly correlated with EMG signal parameters of two of the four muscles of the upper limb—PL and APL. It was shown that there are some groups of recurrent parameters—PDR, PDR regression, mean muscle tension during contraction and skewness, i.e. coefficients carrying different informative values. According to the results, the most challenging for the participants were trials 1 and 3. The first was a kind of introduction to the study, while the third was a slowed-down pace, which could cause anxiety in the subjects and require even more involvement. Positive correlations were obtained, which meant a directly proportional relationship between EMG parameters and reaction time parameters. This may indicate the subjects’ greater nervousness, caused by the situation when they were more aware that they were out of sync with the set rhythm, causing them to generate more and more force during a single contraction^55^. This phenomenon may also be related to greater focus, as the FFT test using pseudosynchronisation is a problematic test requiring subjects’ involvement. Therefore, in the future, it would be worthwhile to include other physiological signals to monitor subjects’ emotions, both positive and negative (electrodermal activity signal or heart rate variability), and eliminate any related artefacts from the EMG signal^56^. In analysing the correlation of EMG parameters with time differences between successive synchronisation errors, correlations between the statistical parameters of time differences and the EMG parameters of two muscles—PL and APL—were also shown. However, there were far more correlations than for synchronisation error analyses. Both direct and inverse correlations were obtained for time differences. The longer the time of a single contraction of the APL muscle, the smaller the differences between successive synchronisation errors—this may indicate the repetitiveness of the movements performed and better synchronisation. A longer contraction was generated at a slower rate, which may suggest ease of adaptation to the set rhythm. It follows that the most significant informational value of the EMG signal should be obtained in the slowed trial, as a greater number of EMG parameters correlate in this approach, allowing the conclusion that the rate of inflicted stimuli may be more important than the reaction time itself in terms of physiological response. Different parameters correlate for individual muscles to different degrees, but in a certain way, they repeat, which again allows us to identify a group of relevant EMG parameters. This correlation will make it possible at a future stage of the design of the expert RAS4NoW system to obtain a detailed description of the subject’s condition—it will be known how a given parameter should behave in subsequent trials if a specific value decreases, then the rate of infliction of metrorhythmic excitations should be increased, decreased or maintained. The specified parameters will allow personalisation of the system and adjustment of the rate of infliction of stimuli in an entirely objective way, rather than in a subjective way as before, where the rate was chosen by the subject. The obtained results of the correlation of EMG parameters and the rate of infliction of stimuli confirm this thesis. The correlation between the achievement of the time of maximum contraction value and the WA parameter is important, as it confirms the necessity of monitoring the PL and APL muscles.

Correlation of EMG signal parameters with synchronisation error parameters and differences in successive error values, during the HST test, in successive trials was carried out in an analogous way. For reaction time analyses, it should be noted that significant correlation coefficients were obtained for three muscles of the lower limb—GL, GM and SL. Both time and frequency parameters correlated; however, there were far fewer correlations than for the upper limb. The least information about the physiological state of the subject’s muscles in relation to reaction time was obtained in trial two, again indicating that trials 1 and 3 are more relevant in analysis and more difficult for the participants, similar to FTT. The strong negative correlations show that the correlations are opposite to those in the FTT. The HST was a simpler, less demanding test and did not stress the test group as much. However, it involved more muscles. The relevant parameters were both time and frequency ratios. For the GL muscle, the greater the value of synchronisation error, the fewer outliers in the obtained error results in trial 1—GL, GM and SL, with a significant predominance of strong inverse-propensity correlations. In the first trial, correlations were obtained for the GL muscle, in the second for SL, and in the third—for GL and GM. Different muscles were engaged at different rates. The varied response of the difference correlation analysis may be due to morpho-functional differences in the analysed muscles and their fatigue observed in successive trials, as well as different functions of the analysed muscles and, in particular, differences in motor cortex control for selected muscles^57^.

The last step of the conducted analysis involved verifying the correlations of the rate of inflicted stimuli and EMG parameters from individual muscles in the HST test, in which strong negative correlations were also obtained, but only for two muscles—GL and SL. The significant parameters coincide with the correlations shown above. For the SL muscle, the strongest inverse correlations were obtained with the regression parameters of EMG coefficients, which means that the higher the rate, among other things, the lower the maximum values of individual contractions and the lower the variability of these maximum values in successive repetitions of the heel stomp during the HST.

Precise timing, as determined by sensorimotor synchronisation, is crucial for various activities. To the best of the authors’ knowledge, there are no similar studies to the ones presented in our article, which compare the values of individual muscle tension during a synchronisation and pseudo-synchronisation test using metrorhythmic stimulation while additionally taking into account the social data of the subjects. The literature also says that there is also a lack of fully functioning and reliable systems that allow the personalisation of therapy based on a broad spectrum of physiological data and the detection of movement patterns specific to the subject while using metrorhythmic stimuli^58^. Nevertheless, this area is attracting the interest of a growing number of researchers. The aforementioned personalised systems are a significant area of research not only because of the advancement of technology but especially because more and more people require rehabilitation due to musculoskeletal dysfunction and an ageing population^59^. Based on the analyses, it has been proven how important it will be to consider musicality since the ability to perceive rhythm affects the improvement of spatiotemporal parameters of gait^60^. However, most of the differentiating characteristics were shown for the case of group division regarding the fact of undertaking musical activities. This means that consciously undertaking activities to improve one’s musical skills constantly is probably more important in achieving better synchronicity test results than simply getting a musical education (at various levels). Therefore, summing up the results obtained, it should be concluded that it will be crucial to consider not so much musical education as the musical activity itself undertaken in recent times, which is confirmed in the literature. Passive articulation of music and overt learning (acquiring musical education) significantly affect music perception^61^. According to a study by Matthews et al., more important in the perception of metrorhythmic stimulation and pacing during guided exercises will be the musical experience itself and exposure to music than playing a specific instrument^62^.

In the future, using the results discussed and developing the target RAS4NoW system, it will be crucial to extract such parameters with the greatest informational value, considering the rate of metrorhythmic stimulation inflicted and the beneficiary’s music education. The use of detailed analyses of synchronisation errors can contribute to the development of methods to improve the rehabilitation process of subjects with motor dysfunction, and this will contribute to the development of an expert system that considers personalised musical preferences.

## Conclusions

Using synchronisation and pseudosynchronisation with metrorhythmic stimuli is becoming a main issue in gait rehabilitation. It has been proven that not musical education but musical activity has a significant impact on the ability to receive (synchronisation ability) and perceive (physiological response) metrorhythmic stimuli at different rates. Based on the results, it was shown that there is a relationship between: (1) musical education and the values of synchronisation errors in HST, (2) the values of synchronisation errors and muscles activity parameters, depending on the rate of stimulation both in HST and FTT, (3) muscles activity depending on the rate of stimulation both in HST and FTT. The selection of relevant muscles from the upper and lower limbs made it possible to determine the areas of analysis and, consequently, the relevant features of the EMG signal. Developing a holistic system based on fundamental knowledge will allow the elaboration of an intervention that, in addition to rehabilitation, will aim to personalise and regulate the state of use to a level that can in the fastest possible recovery.

## Data Availability

The data sets generated and analysed during the current study are not publicly available because those are sensitive data from a specific group. The data sets are available from the corresponding author upon reasonable request.
